# Revealing key role of T cells in neurodegenerative diseases, with potential to develop new targeted therapies

**DOI:** 10.1515/tnsci-2022-0329

**Published:** 2023-12-31

**Authors:** Haofuzi Zhang, Xiaofan Jiang

**Affiliations:** Department of Neurosurgery, Xijing Hospital, Fourth Military Medical University, Changle West Rd. 127, Xincheng District, Xi’an, Shaanxi, 710032, China; Institute of Neurosurgery of People’s Liberation Army of China (PLA), PLA’s Key Laboratory of Critical Care Medicine, Xijing Hospital, Fourth Military Medical University, Xi’an 710032, China

**Keywords:** T cell, neurodegenerative disease, therapy

## Abstract

David M. Holtzman and his team at the University of Washington School of Medicine have made breakthroughs in their research on neurodegenerative diseases. They discovered that the infiltration of T cells into the brain, instigated by activated microglia, is a critical factor in the progression of tauopathy. The groundbreaking findings were published in Nature on March 8, 2023. This research delineates a pivotal immune hub linked to tauopathy and neurodegeneration; a complex interplay involving activated microglia and T cell responses. This discovery could potentially become a target for developing therapeutic interventions for Alzheimer’s disease and primary neurodegeneration.

It is notable that over 20 experimental therapies focused on the immune system are currently in clinical trials for Alzheimer’s disease (AD), underscoring a growing recognition among scientists of the crucial role the immune system plays in contributing to brain damage, which in turn leads to memory loss, confusion, and other debilitating symptoms [1]. Currently, a significant number of AD drugs emphasizing on immunity are primarily targeting microglia [2], the naïve immune cells in the brain. These cells, when wrongly activated or at the inappropriate time, can turn harmful, resulting in damage to brain tissue.

In a recent article titled “Microglia-mediated T cell infiltration drives neurodegeneration in tauopathy” published in Nature, researchers from Washington University School of Medicine illustrated that microglia can communicate with T cells to instigate neurodegeneration in the brain. After studying mice with AD-like brain damage caused by tau protein, the scientists determined that microglia can draw cytotoxic T cells to the brain. Targeting the interaction between microglia and T cells could be a potential therapeutic strategy for neurodegenerative diseases (Figure 1). These research findings suggest that targeting T cells may be a viable alternative approach to treat neurodegeneration and other tau protein-related diseases [3].

**Figure 1:**
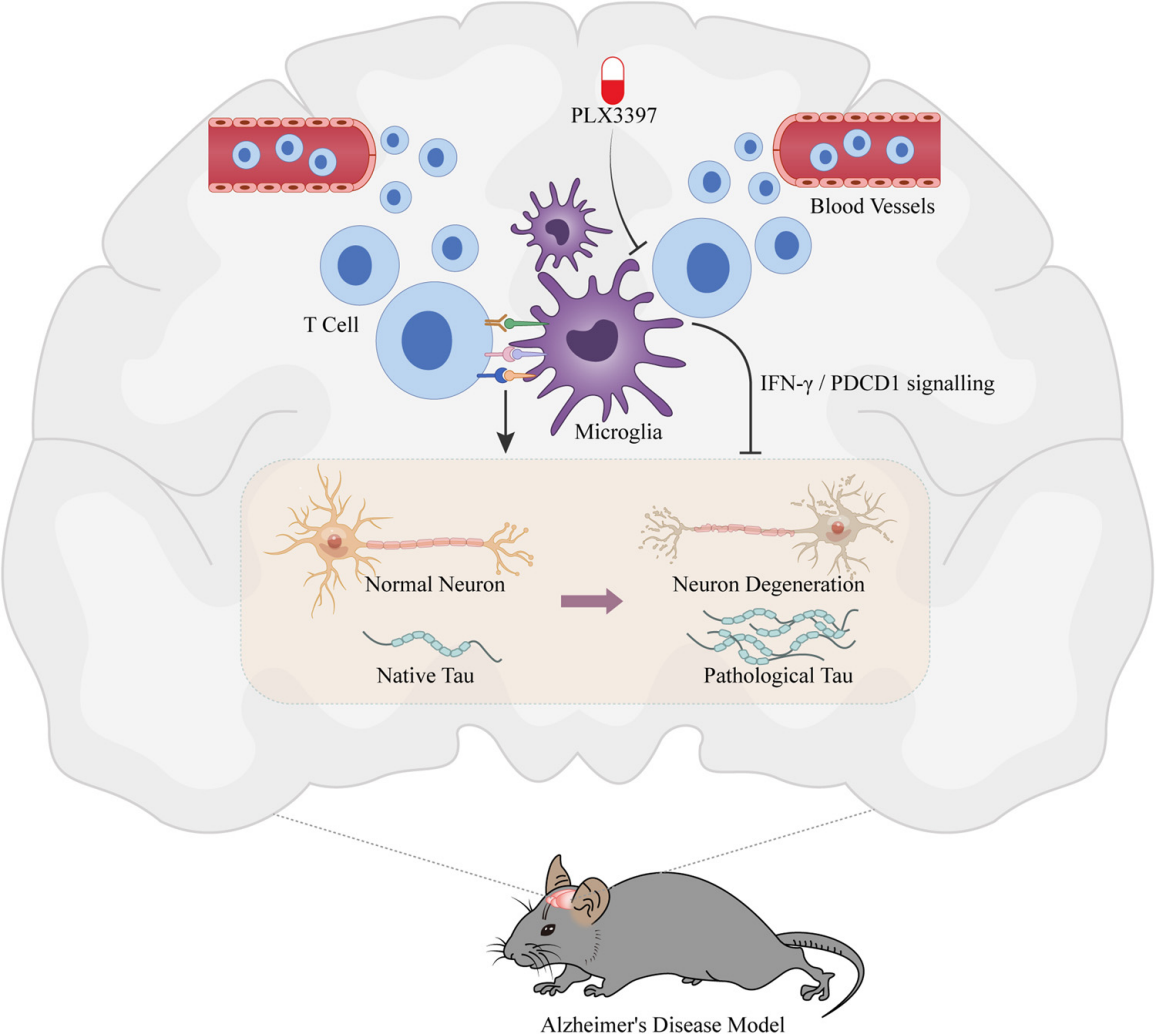
Schematic figure of the research. The research revealed the mechanisms of the neuroimmune microenvironment in AD and investigated the causes of nerve damage, thereby offering a fresh perspective and new methods for interventions in neurodegenerative diseases.

The recently discovered research findings could potentially bring about substantial advancements in the treatment and management of AD and other tau protein-related diseases. While it has been established that T cell levels escalate in the brains of patients suffering from these diseases, the specific role T cells play in causing neurodegeneration remained elusive. This novel research indicates that microglia, in conjunction with T cells, can instigate neurodegeneration and that T cells, when targeted, could be a revolutionary regimen for designing effective therapies. It is worth highlighting that T-cell targeting drugs such as Fingolimod are already employed for other autoimmune disorders affecting the brain and spinal cord, like multiple sclerosis [4]. If these drugs can prove to demonstrate protective effects in animal models of AD and other tau protein diseases, it is highly likely they could progress into clinical trials for treating these conditions.

The progression of AD is typically segmented into two phases [5]. The initial phase is characterized by the formation and accumulation of β-amyloid (Aβ) plaques in the brain over many years, without noticeably impacting brain health. However, during the second stage, the accumulation of tau protein commences, marking a drastic decline in the patient’s condition. This is where brain atrophy and cell death start to occur, resulting in neurodegeneration and cognitive impairments such as memory loss and difficulties in thinking. Extensive research has been conducted by scientists to investigate the role of microglia in the development of AD [6–8]. With the buildup of amyloid plaques, these cells become active and start to dysfunction. As tau protein amasses, the malfunctioning of microglia intensifies neurodegeneration and expedites the progression of the disease [9].

Microglia play an integral role as cellular components within the central nervous system (CNS), partaking in both physiological and pathological processes [10]. They contribute substantially to aspects such as development, learning, memory, as well as neurodegenerative diseases, and fall under the umbrella of the naïve immune response. Considering that the CNS is classified as an “immune privileged” organ, it raises intriguing questions. Is there an adaptive immune response that operates within the CNS, and if present, what would be its biological implications? Does any interaction occur between the naïve immune response and the adaptive immune response? Additionally, could the immune response homeostasis of the CNS be disrupted in a disease environment, thereby contributing to the disease’s onset and progression?

Researchers ventured to investigate the significant role that immune cells in the brain play in the process of neurodegeneration. Their focus was on analyzing the characteristics of these immune cells within the brains of genetically modified mice, simulating various facets of human AD. Their objective was to discern shifts in the immune cell population during the disease’s progression. This intriguing exploration entailed a systematic analysis of two mouse models of AD, namely, Aβ and Tau. This was coupled with the examination of brain samples from clinical AD patients. Their findings exposed that abnormal naïve and adaptive immune responses associated with nerve fiber tangles are the principal drivers of neuron death and brain atrophy. They further discovered that targeting these abnormal naïve and adaptive immune responses could effectively inhibit neuron death and brain atrophy, thereby substantially mitigating the pathological phenotype of nerve fiber tangles.

In addition, researchers garnered a wealth of immune cells from two AD mouse models, Aβ and Tau. Through single-cell expression profiling, they meticulously analyzed the full gamut of characteristics presented by immune cells in relation to two pathological changes, Aβ and neurofibrillary tangles. This analysis was conducted without any bias. Unexpectedly, they discovered that in brain areas such as the hippocampus and piriform cortex of the Tau AD mouse model – which presented with brain atrophy – there were not only substantial changes in microglia, the naïve immune cells, but also a multitude of adaptive immune cells, chiefly characterized by T cells. These were found to be densely populated around neurons with neurofibrillary tangles and showed a significant positive correlation with brain atrophy. APOE, a crucial risk gene for AD, was seen to have a higher degree of recruitment amongst T cells in mice with the high-risk subtype of AD gene, namely APOE4. Researchers discovered a reduction in T cells and a depreciation in microglia activation following APOE knockout. It emerged that APOE not only presides over lipid metabolism but equally performs a critical function in the neuroimmune response. Crucially, the study of clinical brain samples from AD patients substantiated a positive correlation between T cell infiltration into the brain parenchyma and the presence of neurofibrillary tangles, as well as the severity of AD.

The research also revealed a positive feedback loop between microglia and T cells. Activated microglia trigger T cell infiltration, which in turn encourages the transformation of T cells from an uncommitted state to either an activated or an exhausted state. The data from a single-cell analysis of T cell surface receptor (TCR) immunoassays showed that T cells undergo specific TCR enrichment after being recruited to the brain parenchyma, indicating that the cells have encountered specific antigen presentation and undergone clonal expansion. By employing the CSF1 receptor inhibitor PLX3397 to deactivate microglia, or by using T cell neutralizing antibodies to eliminate T cells, abnormal naïve and adaptive immune responses were restrained, resulting in a marked decrease in T cell numbers in the brain parenchyma. Consequently, the Tau AD mouse model demonstrated significant improvements in brain atrophy, neurofibrillary tangles pathology, and microglia activation, as well as cognition and memory.

Half a century ago, pathologists identified that T cells, facilitators of adaptive immune responses, could infiltrate the diseased brain parenchyma [11]. However, the cerebral parenchyma has traditionally been considered an “immune-privileged” site with almost no adaptive immune response. This study is the first to reveal that, akin to naïve immunity, adaptive immune response is a vital element of the CNS’s immune reaction. More significantly, the research discloses that the aberrant immune response, composed of naïve and adaptive immunity, is the central driving force behind the pathological alterations of neurofibrillary tangles that lead to neuron depletion and brain atrophy. Interfering with any link in this anomalous immune response could potentially impede or even reverse the latter-stage pathological transformations in AD.

In conclusion, this study underscores a pivotal immune mechanism that involves activated microglia and T cell responses in correlation with tauopathy and neurodegeneration. Such a mechanism could potentially be leveraged as a specific therapeutic target to thwart the commencement of neurodegeneration in patients with AD and primary tauopathy [12].
